# Deconstruction of the discourse authority of scientists in Chinese online science communication: Investigation of citizen science communicators on Chinese knowledge sharing networks

**DOI:** 10.1177/09636625211005106

**Published:** 2021-04-16

**Authors:** Zheng Yang

**Affiliations:** The University of Sheffield, UK

**Keywords:** boundary work, discourse analysis, discourse authority, exclusive legitimacy, online ethnography, online science communication

## Abstract

This article explores science communication and discussion about genetically modified food on Zhihu, the biggest Chinese knowledge-sharing social network, through the methods of online ethnography and discourse analysis. The long-term online ethnography finds a new group emerging: citizen science communicators – those members of the public without a professional scientific background who actively assume the role of communicators. The discoursal and behavioural characteristics of citizen science communicators effectively eliminate the boundaries between scientists and the public, scientific discourse and that of non-scientists, as well as the ‘exclusive legitimacy’ of science and scientists in the online science communication process. By eliminating boundaries and ‘exclusive legitimacy’, the authority of Chinese scientists has also been challenged and deconstructed in online science communication on Zhihu.

## 1. Introduction

The topic of discourse authority has already proven to be a popular research theme in the fields of science communication, public understanding of science (PUS), public engagement with science (PES), and broader Science and Technology Studies (such as Davies, 2014; Myers, 2003; Wynne, 2006). The discourse authority of scientists is considered to be constructed by science and scientists in interaction with other types of knowledge and non-scientist social groups through various discoursal and rhetorical strategies (Gieryn, 1999; van der Hel and Biermann, 2017). For this reason, the authority of scientists has also not been treated as inherent and unchangeable (Myers, 2003). The monopoly of discourse power and the mastery of authority by scientists are also considered to be obstacles that hinder equal dialogue between scientists and the public and effective public engagement with science (Brossard and Nisbet, 2007). Simultaneously, with the expansion of the scope and deepening of the degree of PES, and the empowerment of digital media for public discourse power, the authority of scientists in science communication – especially in the online science communication process – is considered to have weakened to a certain extent (Atkinson, 1998; Myers, 2003; Petersen et al., 2019). But research on the construction and deconstruction of discourse authority of scientists has received little attention in the Chinese context. This study starts by considering the ‘exclusive legitimacy’ of the discourse authority of scientists in the science communication process by analysing a new group in online science communication in China’s digital media environment: citizen science communicators (CSCs). CSCs and their online behaviour and discoursal characteristics will be used to analyse the deconstruction of the authority of Chinese scientists in the Chinese online science communication process, using discussion of genetically modified food (GMF) on Zhihu as example.

## 2. Legitimacy and discourse authority of scientists in the science communication process

Authority refers to the legitimate power that one person or group possesses and practices over another, including giving orders, making decisions and enforcing obedience (Bealey, 1999). Discourse authority refers to the embodiment of such legitimate power in the practice of discourse, meaning that in written and spoken communications, the legitimate power that one person or group holds requires other persons or groups to trust their words to maintain their own privileged position as speakers or communicators (Fisher, 1984). The discourse authority of scientists is mainly reflected in two aspects: ‘public unconditional trust’ and ‘exclusive legitimacy’ (Myers, 2003; van der Hel and Biermann, 2017). It is also often associated with another important concept: the ‘cultural authority of science’, whereby in addition to public knowledge of science, scholars pay more attention to the public’s trust and acceptance in science and its conditions (Bauer et al., 2018; Gauchat, 2011). Scholars believe that the cultural authority of science offers science and scientists a cultural and institutional monopoly on production and communication of credible knowledge which the public unconditionally trust (Gauchat, 2011; Habermas, 1989). Therefore, the cultural authority of science that is concerned with ‘public trust in science’ can be considered as one dimension of the authority of scientists in the science communication process. In other words, when the public no longer unconditionally trusts science or the discourse of scientists in the communication of science-related topics, the authority of scientists is challenged or deconstructed (Wynne, 2006: 211).

In addition to unconditional public trust, the authority of scientists is also reflected in ‘exclusive legitimacy’. In the theory of cultural authority of science, ‘science’ acquires its cultural or societal authority because it is constructed as the only legitimate type of knowledge which can explain reality (Gieryn, 1999). Van der Hel and Biermann (2017: 216) also found that in the governance of scientific affairs, such as environmentally sustainable development, scientists also claim their exclusive consultative legitimacy by stressing that they represent a multiplicity of relevant scientific fields, which further maintains their authority. In the discourse practice of science communication the authority of scientists is also reflected in the construction and maintenance of similar ‘exclusive legitimacy’. Such exclusive legitimacy is mainly represented in three main aspects. First, only scientific discourse is the ‘exclusive’ (or prioritized) legitimate discourse for interpreting science-related issues, even if the issue also has many other dimensions (Jia, 2015). For instance, the topic of genetically modified food is not just related to science – the public is also concerned about its political, economic, cultural and other dimensions (Jia, 2015). However, to maintain their own authority on GMF issues, science and scientists tend to construct GMF as a scientific issue first to ensure that scientific discourse is the exclusive legitimate discourse for interpreting GMF issues (Cui and Shoemaker, 2018; Jia, 2015). Second, only scientists – with their professional background – are constructed as the legitimate communicators of scientific information or knowledge (Davies, 2014; Myers, 2003). Communicators who do not have a professional scientific background, especially in the digital media environment, are often labelled as untrustworthy and even illegitimate in science communication and discussion (Davies, 2014; Felt and Davies, 2020). Especially in China, members of the public without scientific backgrounds who participate in the communication of scientific knowledge are often labelled derogatively as ‘*minke*’ (crank民科), thereby reducing the legitimacy of their participation in science communication, especially as communicators (Yang, 2008). Third, in science communication, only academic discourses with professional scientific expressions are considered legitimate and having trustworthy communication content. Although scholars have emphasized the need to minimize the use of professional terminology and professional scientific discourse techniques when conducting science communication facing the public (Bauer and Bucchi, 2008), the use of professional scientific expressions, such as professional terminology, scientific diagrams and references are still considered to be important sources for identifying whether a text is scientific and credible or not. Therefore, in the discussion and communication of scientific affairs, ‘informative scientific data’ and ‘objective scientific writing style’ are regarded as important signs of the credibility of a text (Bucchi, 2013). Therefore, communication texts written using non-scientific expressions and styles are often regarded as lacking legitimacy and credibility when discussing science-related topics (Myers, 2003). Although the above three aspects are not necessarily acknowledged by science and scientists themselves – and while some have tried to adopt the form of ‘public engagement’ to further gain legitimacy and authority in governance (see van der Hel and Biermann, 2017: 216–217) – these three types of legitimacy for maintaining the discourse authority of scientists have been widely found to exist (Gieryn, 1999; Myers, 2003; Jia, 2015; van der Hel and Biermann, 2017).

These three aspects of exclusive legitimacy ensure the authority of scientists in the discourse practice of scientific affairs and exclude the intervention of non-scientists. This has also been called the ‘boundary work of science’ by Gieryn (1983, 1999). According to Gieryn, by dividing and emphasizing the boundaries between science and non-scientific knowledge, and scientists and groups of non-scientists, scientists emphasize their exclusive legitimate right to interpret reality, produce reliable knowledge and further maintain their authority in society. The boundary work of science has further been believed to be realized through discoursal and rhetorical strategies in the interaction between science and non-scientific knowledge, and between scientists and other social groups (Gieryn, 1983, 1999; Myers, 2003; Scheitle et al., 2018; van der Hel and Biermann, 2017). Therefore, in science communication, discoursal and rhetorical techniques are considered to be the key to constructing the discourse authority of scientists. Because it is not inherent, but rather depends on the discourse for legitimation, the authority of science may be deconstructed via rhetorical and discoursal techniques.

Previous studies on the deconstruction of the authority of scientists have mostly been conducted on the first component of the authority of science, such as ‘the conditionality of the public’s trust in science and scientists’ or ‘the reduction of public’s trust in science and scientists’ (Gauchat, 2011; Huber et al., 2019; Wynne, 2006). Research is limited on the second component, the ‘exclusive legitimacy’ of scientists in science communication, especially in the Chinese context. In order to fill this research gap, in this article I analyze the discussion of genetically modified food on Zhihu as a Chinese example of deconstruction and weakening of the discourse authority of scientists.

## 3. Research objects and methods

### Objects

This study takes the GMF discussion on Zhihu as a research example. Zhihu, founded in 2010, is now the biggest knowledge-sharing network platform (or Q&A platform) in China with more than 220 million daily active users by the end of 2019. There are more than 1000 active science-related sections on Zhihu, which accommodate almost 1.5 million science-related questions, and more than 3 million answers. This makes Zhihu one of the most comprehensive and popular science communication digital platforms in China. Some Chinese scholars believe that on Zhihu, the interaction and dialogue between scientists and the public in the scientific discussions process has been effectively realized (Cao, 2014; Ju, 2019; Yang, 2016). Zhihu is therefore an appropriate site for exploring changes in the discoursal authority of science in the Chinese context.

This study chose GMF as its research topic for the following reasons. First, the topic of GMF is currently one of the hottest scientific topics in China, which has involved many people of different identities in discussions, including scientists, policymakers, government officials, celebrities, journalists and general publics, both online and offline. Because the merits of GMF are still inconclusive, the lack of a definitive ‘answer’ to the subject has attracted much attention and discussion in China (Qi & Zhou, 2010). By 2016, almost 41.4% of the Chinese public were opposed to GMF, with only 11.9% supporting it. This attitude is in sharp contrast with the general attitude of Chinese scientists, who support GMF (Cui and Shoemaker, 2018). Therefore, there are often fierce quarrels between the Chinese public and scientists over the topic of GMF on the Chinese Internet (Jia, 2014). Some Chinese scholars also point out that Chinese scientists only pay attention to scientific knowledge and ignore emotions when conducting science communication or discussions about GMF (Jia, 2014, 2015). And the Chinese public’s demand for non-scientific information, such as economic or political information about GMF, has further created opposition between Chinese scientists and the public (Jia, 2015). Thus, GMF has generated the most popular scientific controversy in China’s digital media environment, in which the public and scientists are widely involved. On Zhihu, the section on GMF is also one of the most active science sections, which attracts more than 300,000 followers. It is active enough to observe and collect data for exploring the online science communication process as well as the interaction between Chinese scientists and the public. Second, focusing on the potential discoursal interaction between Chinese scientists and the public, the fields of traditional ‘hard science’, such as particle physics – with low public impact and mobilization, little controversy among experts, and propelled by visible research institutions – may lead to a deficit-like pattern in which the public is invited and willing to appreciate the spectacle of science’s achievement (Bucchi, 2008: 71). However, an issue such as GMF – touching many publicly relevant themes, including food, safety, biodiversity and resource distribution, with a certain amount of disagreement between experts on the issue, propelled by corporate actors in a highly sensitive context, alert and mobilized to questions of environment and globalization – is unlikely to lead to a deficit of interest with public audiences (Bucchi, 2008: 71). Thus, this topic is suitable for exploring the dynamics of the discourse authority of scientists based on interactions between Chinese scientists and the public.

### Methods

This study mainly draws on online ethnography, the research approach that applies traditional ethnographic research methods to a computer-mediated communication environment (Androutsopoulos, 2008). In online ethnography, the practice of fieldwork has changed from a physical space to a virtual discussion community, which has introduced new media culture such as chatrooms into ethnographic studies (Bogdan and Biklen, 2007). As a research approach, online ethnography can help to find and analyse the interactions and communications between different groups in a community in more depth in the networked environment (Hine, 2000). Online ethnography is an appropriate method for exploring the collective characteristics of the behaviours of a certain group in online environment. There are many ways to conduct an online ethnography, such as following people, views, discourses, groups or behaviours (Marcus, 1995). But since online science communication is mainly reflected at the textual level, the online ethnography in this study mainly focuses on the textual and discoursal level on Zhihu.

According to Hammersley and Atkinson (2007), there are five steps for online ethnography research, which this study follows: (1) defining research issues and survey topics, (2) identifying and selecting communities, (3) community participant observation and data collection, (4) data analysis and interpretation, and (5) composing and showing findings. This study uses the section about GMF on Zhihu as its research community. To conduct participant observation, the author registered a Zhihu account in December 2017 and started to participate in the GMF section from January 2018. The participatory observation lasted almost two years, until October 2019. During the online ethnographic observation, the researcher logged on to Zhihu, entered the GMF section, made observations and wrote observation logs almost every other day. Each time, the observation lasted from 30 minutes to an hour. By the end of the observation, the researcher had observed the community for 219 hours. During the ethnography, the author also proposed two questions and provided four answers in the GMF section on Zhihu (which was established on 17 February 2011). During the first 6 months of the online ethnography, the focus of the observation was to examine and record the users and their behaviours already present in this section, such as questions, answers and numbers of comments and likes. After 6 months, the focus of observation was shifted to the daily changes in this section, such as the introduction of new questions, the provision of new answers, the change in the number of likes and comments, and the change in the users’ attitudes towards a certain answer. In each observation, the author recorded the following information about each question and/or participant: questions, answer content, numbers of likes and comments towards each answer, and personal information of answerers, and so on (for Ethnographic Observation Information Record Form, please see the Supplemental material).

**Figure 1.**
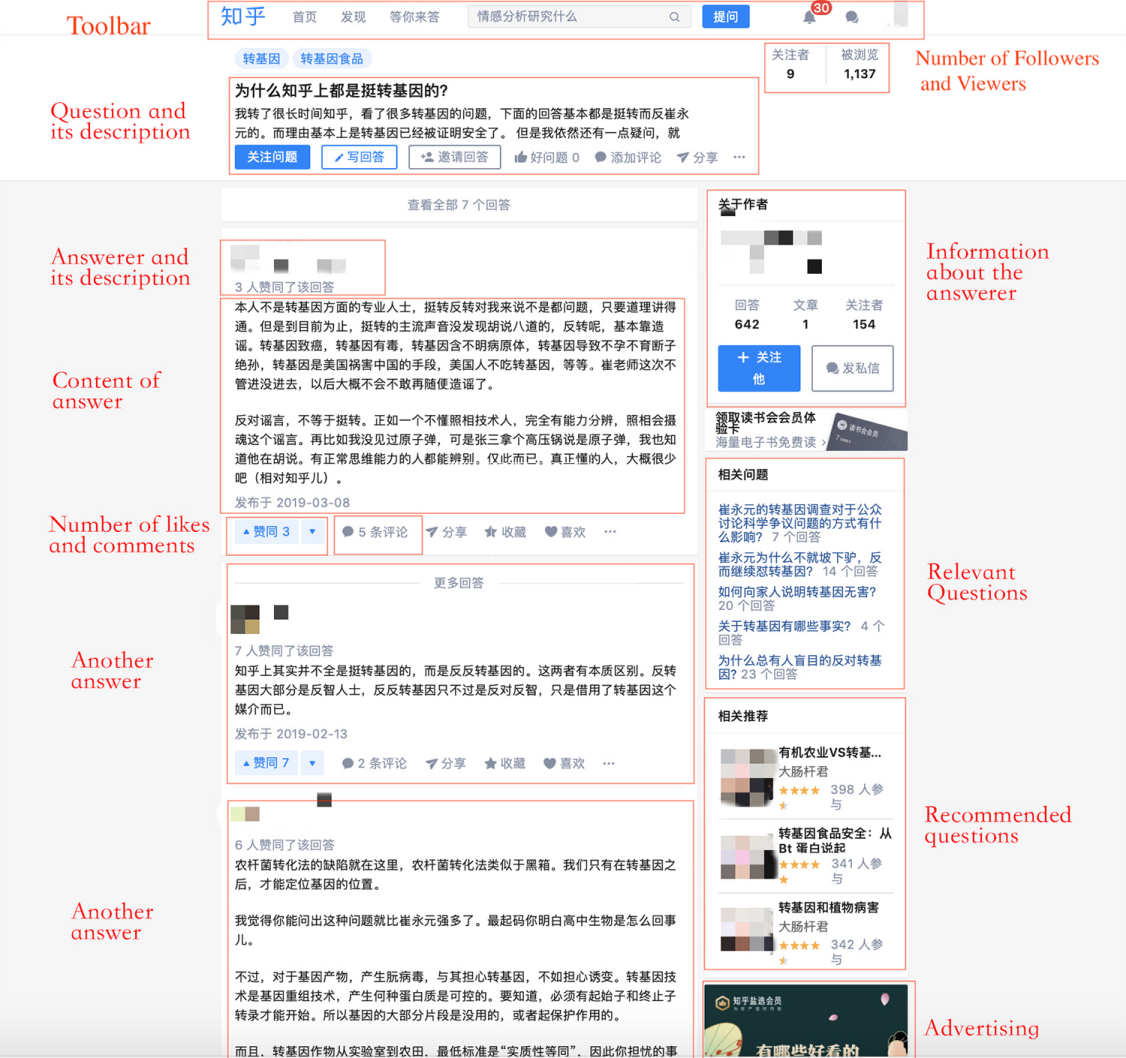
Basic information on Q&A page on Zhihu.

Most of the information above was recorded as text, such as the content of answers, questions and comments, with a description of the users who provided them. Some data changes were also recorded. Figure 1 shows the basic information of the question and answer page of Zhihu, the section observed and the source of recorded information in the ethnography (the usernames and profile photos have been anonymized). The users on Zhihu were divided into three categories – scientists, non-scientists and NULL (non-individual users or identities that were difficult to clarify) – according to both the users’ self-description on their homepage on Zhihu and the content of the answers they provided and questions they asked. Most Zhihu users provide their identity information, such as educational experience and career on their homepage, which was the primary criterion for judging their identity. For those users who did not provide clear identification information, their identities were sought through sending private messages. If valid information about their identity was not provided, those users were marked as NULL.

Alongside the online ethnography, discourse analysis was also used as a complementary method to analyse the recorded data, especially the content of answers. It is valuable to study not only the discourse features of written content, but also the power relationship between different groups, such as scientists and the public, and the dynamics of such relationships in the Chinese digital media environment. In this study, the discourse analysis mainly focused on the linguistic features of the selected samples, such as language structure, presentation style, language norms and genres of the answers provided in the GMF section on Zhihu.

## 4. Findings

### Citizen science communicators and their popularity on Zhihu

Through the 2-year online ethnography of the GMF section of Zhihu, the most notable finding was that a special group emerged: Citizen science communicators. The emergence and activities of this group have challenged the authority of Chinese scientists in online science communication. The following analysis considers this group by exploring their online behaviour and discourse characteristics.

CSCs are defined here as a branch of citizen scientists. The concept of ‘citizen scientist’ originated from the new model of public participation in scientific research, ‘citizen science’ projects, which refer to those scientific projects that actively involve citizens in scientific endeavours to generate new knowledge or understanding (Strasser et al., 2019). In those projects, the concept of citizen scientists refers to those members of the public or volunteers outside of the research project who collect and/or process scientific data as part of a scientific enquiry (Silvertown, 2009). Those volunteers are called citizen scientists because they assume the social responsibilities that originally belonged to scientists: scientific research and knowledge production (Silvertown, 2009). Although this concept originally referred to members of the public who were directly involved in the scientific research process, as the concept developed its connotations gradually expanded to refer to those citizens who actively take on various responsibilities of scientists, including scientific research and other societal science-related responsibilities, such as communicating science-related knowledge (Patel et al., 2016). Therefore, by inheriting the core concept of citizen scientists, the CSCs (who could also be called ‘amateur science communicators’) in this article refers those citizens who have no traditional scientific background or are not currently engaged in any scientific employment but actively undertake scientists’ traditional societal responsibility to communicate and disseminate scientific knowledge or science-related information on digital platforms like Zhihu.

For instance, User *A*’s Zhihu homepage bio reads, ‘Professional in insurance on Zhihu’. However, this user widely participated in discussions on scientific topics such as GMF on Zhihu. Before January 2018, this user had already answered 34 questions about GMF on Zhihu. From January 2018 to October 2019, the period of online ethnography observation, this user further answered 39 questions about GMF, and most of these answers were of high quality, containing a wealth of information. During the observation period, user A’s followers increased from around 10,000 to more than 36,000, and they became the most active communicator with a non-scientific background in the GMF section of Zhihu. For instance, this user answered the question ‘Can genetically modified food really solve hunger in the world?’ In their answers, user A first made the point clear that

Genetically modified (new food technology) is not to solve the problem of hunger, but to increase the population of the earth to a higher level in a more harmonious and environmentally friendly manner, such as from 7 billion to 8 billion, 10 billion or even 20 billion.

They used figures and other evidence from a United Nations World Population Prospects report to prove their points. While not a traditional scientist, they tried to answer a science-related question using secondary data with a basic scientific answer mode using data and resources. Although they had no relevant academic background, their answers still provided rich information and gained many ‘likes’ from other users. Therefore, when such users provide relevant scientific information and answers to other members of the public as ordinary citizens with no scientific background and actively undertake a scientist’s traditional responsibility to disseminate science-related information to others, like *User A*, they can be termed CSCs.

Comparing different types of users on Zhihu, it was found that CSCs are not an unusual phenomenon. Taking the excellent 998 answers^1^ with the most likes on the topic of GMF on Zhihu in October 2019 as examples, only 270 answers were provided by users with scientific backgrounds. In other words, CSCs contributed more than 60% of those excellent answers with the most likes about GMF on Zhihu. These data show that CSCs are highly involved in scientific knowledge-sharing on Zhihu. Further investigation on the topic found that the number 1 active answerer who replied to the most questions in the GMF section on Zhihu was also a CSC.

**Figure 2.**
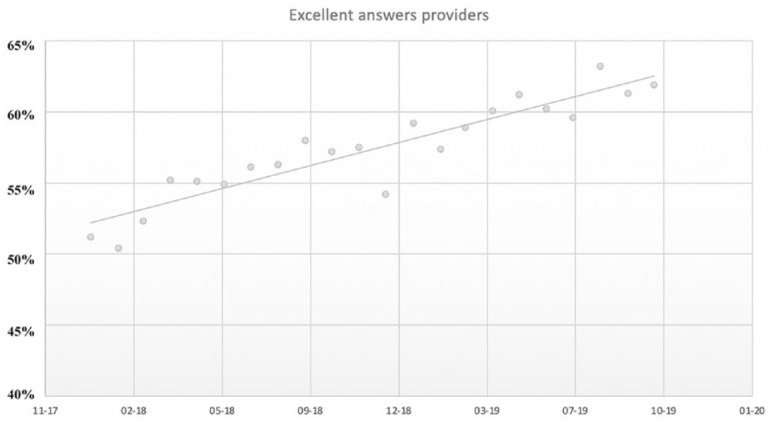
Diachronic changes of the proportion of CSCs during online ethnography period.

The data above suggest that on the scientific topic of GMF on Zhihu, CSCs already play an active role as communicators. Their popularity and the large number of likes received by their answers also illustrates that CSCs’ engagement with the science communication process without a professional scientific background is also accepted by Chinese netizens on Zhihu. Over time, CSCs as a proportion of providers of excellent answers have increased in the GMF section during the almost 2-year online ethnography (Figure 2). The popularity of CSCs also shows that the legitimacy of this group as science communicators on Zhihu has been recognized. Combined with evidence from existing literature, the recognition of CSCs as science communicators has to some extent challenged scientists’ monopoly on legitimacy as science communicators, which will be expected to challenge scientists’ authority in science communication.

### Discourse feature: Using humour – Science communication does not have to be serious

Through the online ethnography and discourse analysis of the answers provided by CSCs, the most significant difference between the science communication led by CSCs and traditional scientists is that the content provided by CSCs has clear humorous tendencies, which is uncommon and considered unsuitable in the traditional science communication.

Science communication (also known as science popularization in China) in the traditional media environment is taken seriously in the Chinese context (Yang et al., 2015) and it is less common for scientists or media occasionally to use humour when conducting science communication. Scientists take pains to communicate correct and objective knowledge to the public through various media platforms. Recently, although many scholars have begun to emphasize the interest and readability of science communication content in taking advantage of digital media platforms (Baram-Tsabari and Lewenstein, 2013), the seriousness of science communication is still deeply rooted in the practice of Chinese scientists. Chinese scholars have found that, even on social media platforms such as Sina Weibo, when Chinese scientists are engaged in personal science communication (independent of media translation), they still tend to use a serious tone (Li and Jin, 2019; Wang et al., 2018). ‘Seriousness’ is already a discoursal boundary between scientists and non-scientists in wider society (Gieryn, 1999) and it is also an important manifestation of scientists’ exclusive legitimacy at the level of content expression in the science communication process mentioned earlier.

On Zhihu, the majority of CSCs’ contributions related to GMF are given in a non-serious manner, for example these two CSCs’ answers:

Genetically modified food is too cheap, Bengong^2^ only buys expensive ones, not the right ones.

Oh, my dear aunt, have you ever heard of space breeding? What? You never heard that before? It was really popular more than a decade ago. Put the seed of the plants on the spaceship, go for a walk-in space, and then come back, take a look. Guess what? Oh my god, that pepper is twice as big as before. Do you think this technology is good?

**Table 1.** Providers of excellent answers providers on the topic of genetically modified food on Zhihu.

	CSCs	Scientists	Null	Total
Excellent answers (up to October 2019)	618 (61.9%)	270 (27.1%)	110 (11.0%)	998 (100%)
Total number of likes received	1,226,769	628,481	23,113	1,878,363

CSC: citizen science communicator.

The humour used in the exaggerated tone or teasing content in these examples would normally be considered by Chinese scientists and scholars as inappropriate for science communication (Li and Jin, 2019; Wang et al., 2018). The use of slang or sayings is also considered to be a means of humorous expression in Chinese, especially enigmatic folk similes (*Xiehouyu*; Rohsenow, 1991). According to Shu (2015), the use of *Xiehouyu* and other folk sayings in Chinese can have ‘humorous’ and ‘smart’ effects. The folksiness and humour of *Xiehouyu* mean that they are considered not serious enough to be used in official settings, such as the traditional science popularization process. However, on Zhihu, such humorous folk sayings and *Xiehouyu* are widely used by CSCs in their science-related answers. For instance,

When you can only eat meat in the Spring Festival, do you care whether the meat you eat comes from free-range pigs or pigs that grew up on feed? Do not take up the bowl to eat, put down the chopsticks and say some dirty words (Behave as a gentleman while the dishes are being served; Swear like a bully when the dinner is over! No treat, no civilization!)

Like using enigmatic folk similes, using broad analogies is also considered to be an unserious form of expression in the Chinese traditional science popularization process. In the view of traditional science popularization in China, science should be accurate, and its purpose is to convey this truth to the public. Although the media occasionally uses some simplifications or metaphors in their ‘science paraphrasing’, such behaviour is likely to be criticized by scientists for damaging the accuracy of science, as Gregory and Miller (1998) have claimed for the British context. However, in the answers provided by these CSCs on Zhihu about the topic of GMF, such ‘unserious’ analogies are widely used:

Risks need to be faced, which is human nature. But if we just wanted to avoid all risks, we would not have fallen from the trees in ancient times. Those who find lions in the grass and those who don’t let people down from the tree are not the same thing.

When it comes to genetically modified food, I think there is a metaphor that makes sense: a group of people walk blindfolded, and a person runs with their eyes open. Which one do you think is safer?

Another form of humorous and non-serious expressions on Zhihu, used by Chinese netizens, is that of dramatic metaphors or analogy, known as ‘shaking clear’ (*doujiling*, a Chinese phrase similar to producing humour; Jin et al., 2017). D*oujiling* expressions usually answer questions in unexpected ways with few sentences, thus creating feelings of humor and amazement. For instance,

The hazards: the IQ of a large group of people is exposed, affecting the money bag (income) of organic food suppliers.

Such forms of expression that do not directly answer scientific questions are also considered unsuitable for use in science communication and the science popularization process. Although the CSCs’ humorous and non-serious answers are not purely scientific, they play a role in conveying certain scientific information and perspectives of thinking about the issue of GMF. In traditional science popularization in China, the content of science communication has long been conducted in a serious style and most of the scientific communication texts written by individual scientists on digital media platform also emphasize the seriousness of science (Chen, 2014). This seriousness has defined the boundaries between credible (led-by-scientists) and non-credible (led-by-non-scientists) science communication in China. The CSCs, with their popularity and widespread acceptance, and their humorous, informal discourse, deconstruct this boundary, and further turn non-serious content into acceptable science communication, challenging the exclusive legitimacy of scientists as the only trustworthy voices in online science communication. The discourse authority of scientists on Zhihu has thus been challenged.

### Discourse and behaviour feature: Multi-perspective discussion – GMF is not just about science

In traditional science popularization or scientific discussion in China, only ‘science’ has the right to interpret issues related to science. Other aspects related to these issues are often suppressed and ignored. Science in China is overwhelmingly understood to mean natural and health science, rather than social or humanities science (Chen, 2013). However, the issue of GMF is not only related to science but also involves many other factors, such as people’s livelihoods, food culture, the economy and politics. In the Chinese context, many non-scientific perspectives on GMF have been ignored while the relevant science is over-magnified. According to Chinese scholars’ research, most of the current GMF reports in China, whether published in traditional or digital media, are mostly drawn from a scientific point of view, explaining the scientific principles of GMF. Most of the figures who appear in such reports are scientists in biology, agriculture or related natural science fields rather than experts from other disciplines (Chen, 2013; Jiang, 2018). However, just as Helliwell et al.’s (2019) analysis of the non-scientific dimensions of the discourse of genome editing in the Western context shows, the scientific ‘monopoly’ on the discussion and communication of GMF topics has been deconstructed to some degree in CSCs’ discussion on Zhihu.

Although the CSCs’ answers to questions about GMF on Zhihu involved some scientific content, on the whole they also approached the topic from different perspectives. For instance, from the perspective of policy, one CSC said the following:

The food issue is a national strategic issue, as is the strategic position of oil in industry. Grain reserves and petroleum reserves and strategically prominent in any country. To achieve a basic well-off in China, food must also be basically self-sufficient . . . You and me cannot do an experiment and write an article to prove scientifically that GM is harmless. But we need to understand the value of GM from other perspectives . . . According to the data from the Development Research Center of the State Council, by 2020, the food demand will reach 548.87 million tons based on 1.43 billion people. By then, China's domestic food supply gap will be 40 million to 50 million tons. Genetically modified crops can just fill such a food supply gap.

Other CSCs answered from a historical perspective:

The Donglin Party in Chinese history is precisely the group of people who were most active and interested in the scientific knowledge brought by Christians and missionaries in the late Ming Dynasty. It is estimated that they will also take the initiative to accept genetically modified food, if they were in today.

Some CSCs used the perspective of culture:

Many Chinese people accept Chinese medicine, maybe because their names are romantic and friendly, such as Angelica (Danggui), Sedum (Jingtian) and Xuchangqing. They all have a kind of Chinese culture in them. Especially Chinese culture has a special good feeling for plants growing out of the land. But for synthetic western medicine, most of them have a cold name, such as ‘azole’ or ‘amine’, which are difficult to be directly accepted at a cultural level by Chinese.

And still other CSCs employed the perspective of philosophical logic:

Cui Yongyuan made a logic mistake: selective bias in the selection of interview subjects. Judging from his edits, he basically selected only those who were opposed to GM, and most of those who agreed to GM were not interviewed by him.

The CSCs broaden the discussion of GMF to include other dimensions. The popularity and widespread acceptance of citizen science communicators shows that users on Zhihu also accept this kind of interpretation of GMF from non-scientific perspectives, which further indicates that scientific discourse led by scientists is no longer the only legitimate way to communicate or discuss GMF on Zhihu. Thus the discourse authority enjoyed by scientists has to some extent been challenged and deconstructed.

## 5. Discussion and conclusion: Deconstruction of scientists’ discourse authority on Zhihu

According to Thomas Gieryn’s boundary-work theory, the authority of scientists comes from the construction of the boundaries between scientists and the public, between scientific discourse and non-scientific discourse (Gieryn, 1983, 1999). In other words, breaking down the authority of scientists lies in the deconstruction of such boundaries. An earlier examination of existing literature proposed three expressions of discourse authority of scientists in science communication based on such boundaries: first, only scientific discourse is the exclusive legitimate discourse for interpreting science-related issues, even if the issue also has many other dimensions (Jia, 2015); second, only scientists with a professional background are the legitimate communicators of scientific information or knowledge (Davies, 2014; Myers, 2003); and third, only academic discourse with professional scientific expressions is legitimate and trustworthy (Myers, 2003). On Zhihu, the emergence of CSCs deconstructs the boundaries between scientific discourse and non-science discourse, scientists and the public.

First, scientists are not only no longer the only accepted legitimate communicators – they are no longer even the main communicators in GMF science communication and discussion on Zhihu. The emergence and popularity of the CSCs shows that members of the public without a professional scientific background can also be accepted as legitimate scientific communicators by collecting, integrating and transmitting scientific information on Zhihu. Therefore, the boundary is blurred between ‘scientist-communicators’ and ‘public-audiences’. From the perspective of boundary-work theory, the double deconstruction of exclusive legitimacy and role boundaries based on the emergence and popularity of CSCs on Zhihu challenges the authority that Chinese scientists enjoyed in the process of science communication.

Second, traditional science communication is considered to be serious and purely scientific, which not only excludes more possibilities of discourse, but also clearly constructs the boundary between ‘orthodox’ and ‘unorthodox’ science communication, especially in the Chinese context (Ren and Zhai, 2013). On Zhihu, science communication is no longer merely serious and purely scientific – non-serious and multi-perspective discourses are accepted by other users as a reasonable part of science communication.

The emergence and popularity of CSCs, and their challenge of the discourse authority of Chinese scientists could have a profound impact on China’s online science communication. First, the deconstruction of the exclusive legitimacy of science or scientist and the breaking of the identity boundary between ‘scientist-as-communicator’ and ‘public-as-audience’ could elicit more public participation and interaction in online science communication and discussion. A paternalistic style and one-way linear communication process are considered one impediment to effective public participation and interaction in science communication and discussion (Huckin, 2002; Bucchi and Trench, 2014; Myers, 2003). CSCs communicate in a way that resonates with the public’s identity and discourse habits, which provides more possibilities for online science communication to be interactive and accessible. However, the popularity of CSCs also has potential dangers. Users without professional scientific knowledge backgrounds cannot guarantee the accuracy of the information that they collect, understand and disseminate. Many rumours or misunderstandings about GMF come from these public communicators (Ji et al., 2019). Therefore, we must maintain a clear understanding of both the potential positive and negative effects that CSCs may have on the science communication system.

In conclusion, this study used online ethnography and discourse analysis to explore online science communication and discussion on the genetically modified food section of Zhihu, the biggest Chinese knowledge-sharing network. The 2-year online ethnography identified a new group emerging: CSCs. CSCs are those public users without a scientific background who actively assume the social responsibility of communicating scientific knowledge and information. Through exploring the characteristics of this new group, it was found that science communication on Zhihu no longer needs to be serious and formal and can look at science issues from historical, political and cultural perspectives. The emergence and popularity of CSCs and their discoursal and behavioural characteristics on Zhihu effectively deconstructs the boundaries between scientists-as-communicators and the public-as-audiences in the science communication process, considered to be the main source of discourse authority of scientists. Therefore, it can be inferred that the discourse authority of Chinese scientists has been challenged and deconstructed by the public, especially CSCs, in the Chinese online science communication process.

## Supplemental Material

sj-pdf-1-pus-10.1177_09636625211005106 – Supplemental material for Deconstruction of the discourse authority of scientists in Chinese online science communication: Investigation of citizen science communicators on Chinese knowledge sharing networks Click here for additional data file. Supplemental material, sj-pdf-1-pus-10.1177_09636625211005106 for Deconstruction of the discourse authority of scientists in Chinese online science communication: Investigation of citizen science communicators on Chinese knowledge sharing networks by Zheng Yang in Public Understanding of Science

## Notes

1. ‘Excellent answers’ (精品回答) are selected by the Zhihu platform in each topic section based on the numbers of likes, comments and content quality of the answers combined with comprehensive artificial and algorithmic measurement. The selected excellent answers all have higher quality content, contain more information and have the greatest number of likes and comments in the entire topic section. Therefore, they are also the most popular and influential answers in each topic section.

2. Bengong is a self-address used by ancient Chinese court ladies, which has become popular thanks to several Chinese television series, such as *Empresses in the Palace* (*Legend of Zhenhuan*) in recent years.
