# Supplementary material for: Deconstruction of the discourse authority of scientists in Chinese online science communication: Investigation of citizen science communicators on Chinese knowledge sharing networks
Source: Public Underst Sci. 2021 Apr 16;30(8):993–1007. doi: 10.1177/09636625211005106 (PMC8551423; doi:10.1177/09636625211005106)
Supplement: sj-pdf-1-pus-10.1177_09636625211005106 – Supplemental material for Deconstruction of the discourse authority of scientists in Chinese online science communication: Investigation of citizen science communicators on Chinese knowledge sharing networks [file sj-pdf-1-pus-10.1177_09636625211005106.pdf]

SUPPLEMENTAL MATERIAL

Deconstruction of the Discourse Authority of Scientists in Chinese Online Science Communication: Investigation of Citizen Science Communicators on Chinese Knowledge Sharing Networks

Zheng Yang

Ethnographic Observation Information Record Form

No.		
Questions:		
Record date		
Answer	Content	
	Perspective	
	Number of likes	
	Number of comments	
	Release date	
	Whether modified (modified date, modified content)	
Answerer	Self-description	
	Educational background	
	Employment	
	Identity	
	Number of questions answered	
	Number of followers	
